# Navigating the Evolution of Digital Twins Research through Keyword Co-Occurence Network Analysis

**DOI:** 10.3390/s24041202

**Published:** 2024-02-12

**Authors:** Wei Li, Haozhou Zhou, Zhenyuan Lu, Sagar Kamarthi

**Affiliations:** Department of Mechanical and Industrial Engineering, Northeastern University, Boston, MA 02115, USA; li.wei10@northeastern.edu (W.L.); zhou.haoz@northeastern.edu (H.Z.); lu.zhenyua@northeastern.edu (Z.L.)

**Keywords:** digital twins (DT), keyword co-occurrence network (KCN), artificial intelligence (AI), sensors, scientometric analysis

## Abstract

Digital twin technology has become increasingly popular and has revolutionized data integration and system modeling across various industries, such as manufacturing, energy, and healthcare. This study aims to explore the evolving research landscape of digital twins using Keyword Co-occurrence Network (KCN) analysis. We analyze metadata from 9639 peer-reviewed articles published between 2000 and 2023. The results unfold in two parts. The first part examines trends and keyword interconnection over time, and the second part maps sensing technology keywords to six application areas. This study reveals that research on digital twins is rapidly diversifying, with focused themes such as predictive and decision-making functions. Additionally, there is an emphasis on real-time data and point cloud technologies. The advent of federated learning and edge computing also highlights a shift toward distributed computation, prioritizing data privacy. This study confirms that digital twins have evolved into complex systems that can conduct predictive operations through advanced sensing technologies. The discussion also identifies challenges in sensor selection and empirical knowledge integration.

## 1. Introduction

The concept of digital twins has evolved beyond its original role in product lifecycle management [1] and become an essential element in the digital transformation across various sectors. The digital twin applications typically involve the creation, utilization, and sustainment of a virtual counterpart of a physical system, facilitating real-time, two-way data exchanges [2]. Digital twins enhance human-machine interactions and inter-machine communications. They dynamically and behaviorally mirror their physical counterparts, integrating both raw and processed data to reflect real-world conditions accurately. While proactive development of digital twins is advocated for optimal integration, retrofitting remains a common practice for existing systems [3].

**Figure 1:**
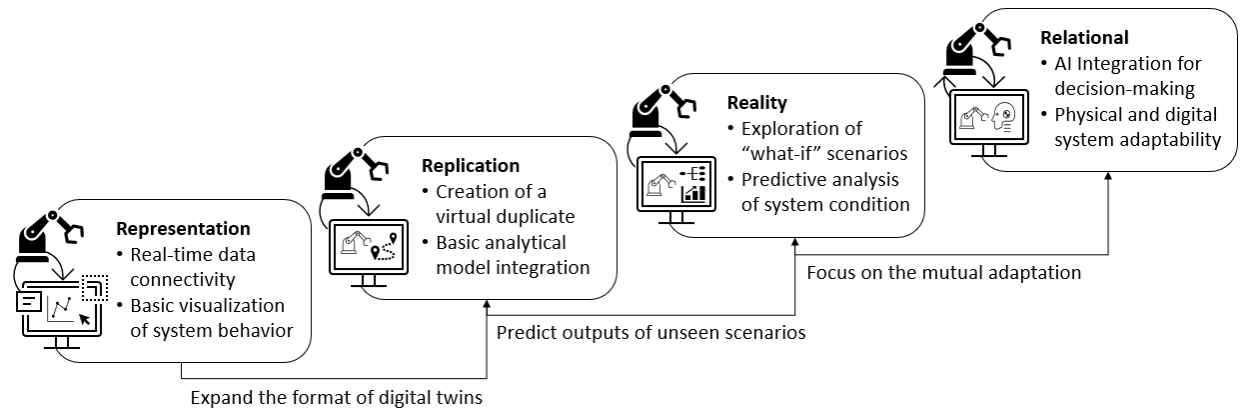
Four levels of functionalities of digital twins.

Digital twins are categorized into four functional levels: representation, replication, reality, and relational [4], as shown in Figure 1. The foundational level, *representation*, focuses on data collection and physical system representation. The form of digital twins at this level is usually real-time data connectivity and visualization. A virtual model is created at the *replication* level to duplicate the physical system and produce the same outputs as the physical system. With the aid of cutting-edge simulation approaches, virtual models have been capable of monitoring and controlling industrial systems with more complex configurations [5]. Digital twins at this level are usually equipped with basic analytical models that can analyze and predict system conditions given existing scenarios. The *reality* level expands the digital twin’s capabilities for exploratory “what-if” analyses, enabling predictions for hypothetical changes and scenarios. The most advanced level, *relational* function, equips digital twins with machine learning models, providing insights that can be acted upon to optimize the physical system performance. Digital twins at this level achieve a seamless close-loop bidirectional data flow and integration between the physical and virtual realms.

Smart technologies such as the Internet of Things (IoT) and Cyber-Physical Systems (CPSs) have been thoroughly studied before the emergence of digital twins. The IoT is a network of physical objects with embedded sensors and other technologies that connect and exchange data with other devices [6]. On the other hand, CPSs are complex systems that integrate the cyber world and the physical world through computing, communication, and control [7]. Although CPSs and digital twins seem similar in definition, they differ in their primary focuses. Digital twins focus on creating a comprehensive virtual model that mimics and predicts the behavior of its physical counterpart [8]. In contrast, CPSs focus on real-time control and the ability to respond to physical states, often through direct sensor and actuator involvement.

Digital twins rely on the same or similar the IoT and CPS-enabling technologies. Because of this, digital twins, as with the IoT and CPSs, have expanded beyond manufacturing and into various other industries. The sensing ecosystem, which includes traditional physical sensors, advanced data analytics, and processing platforms, is central to the expansion of digital twins [9]. The sensing ecosystem captures and interprets the vast streams of data generated by the IoT and monitored by CPSs, forming the backbone of digital twin functionality and setting the stage for the vital role of sensors in digital twin-supported integrated systems.

Sensors, commonly represented by physical devices such as accelerometers and temperature gauges, are the fundamental interface between the physical and digital worlds. The basic structure of a sensor moduleserves to convert measurable physical phenomena into data streams for analysis and application in digital systems [10]. Yet, the definition of sensors has expanded in today’s interconnected environment. Today, a sensor can be anything that translates real-world variables into data, ranging from social media posts that gauge public sentiment to medical tests that provide insights into a patient’s health. This broader interpretation of sensors enables sensors to serve as the primary medium for data flow and analysis across various applications.

Table 1 features a collection of papers that review digital twin applications in various areas highlighting their contributions and identifying gaps and opportunities. This work adds quantitative research trend analysis to the current digital twin review landscape with Keyword Co-occurrence Network (KCN) analysis. KCN analysis is a tool to analyze the research landscape from the metadata of literature [11]. This method allows us to thoroughly examine and interpret the extensive range of digital twin research.

This review paper serves digital twin researchers and architects. The KCN method reveals the interconnectedness of knowledge components, concepts, technologies, and methodologies in digital twin research, aiding researchers in identifying emerging trends and under-researched areas. For digital twin architects, the KCN analysis provides information on the practical application of sensors and other advanced digital twin technologies. It can assist them in making informed architectural decisions and understanding the evolving landscape of digital twin applications in relation to Cyber-Physical Systems.

The remainder of this work is structured as follows. In the methodology section, we explain the process of KCN analysis and its implementation in the context of the digital twin literature. In the results section, we present the analysis results, which include the temporal analysis of research trends, mapping of sensing technology to application fields, and detailed analysis of digital twin applications in various fields. These insights are presented with a series of visualizations and tables that illustrate the interconnected landscape of digital twin research. In the discussion section, we explain the implications of these findings and conclude by reflecting on the challenges currently being faced and the potential paths for the future development of digital twins.

## 2. Methods

This study applies KCN analysis to investigate research trends in digital twin technology. The methodology includes a temporal KCN analysis to identify trends over time and a detailed review of principal application categories. This section outlines the process for article collection, keyword extraction, KCN construction, and network evaluation metrics.

### 2.1. Article Collection and Screening

This study began with a thorough search for literature related to recent advancements in digital twins. We queried literature from Engineering Village, IEEE Xplore, and PubMed. Engineering Village and IEEE Xplore ensure comprehensive coverage in engineering-related subjects, and PubMed provides additional coverage of medical literature. We selected articles that contain the terms “digital twin” OR “digital twins” in the metadata (title, keywords, and abstract). After narrowing the search to peer-reviewed journal articles and conference proceedings published in English from 2000 to 2023, we identified a total of 9639 papers and downloaded the metadata of this article collection.

**Figure 2:**
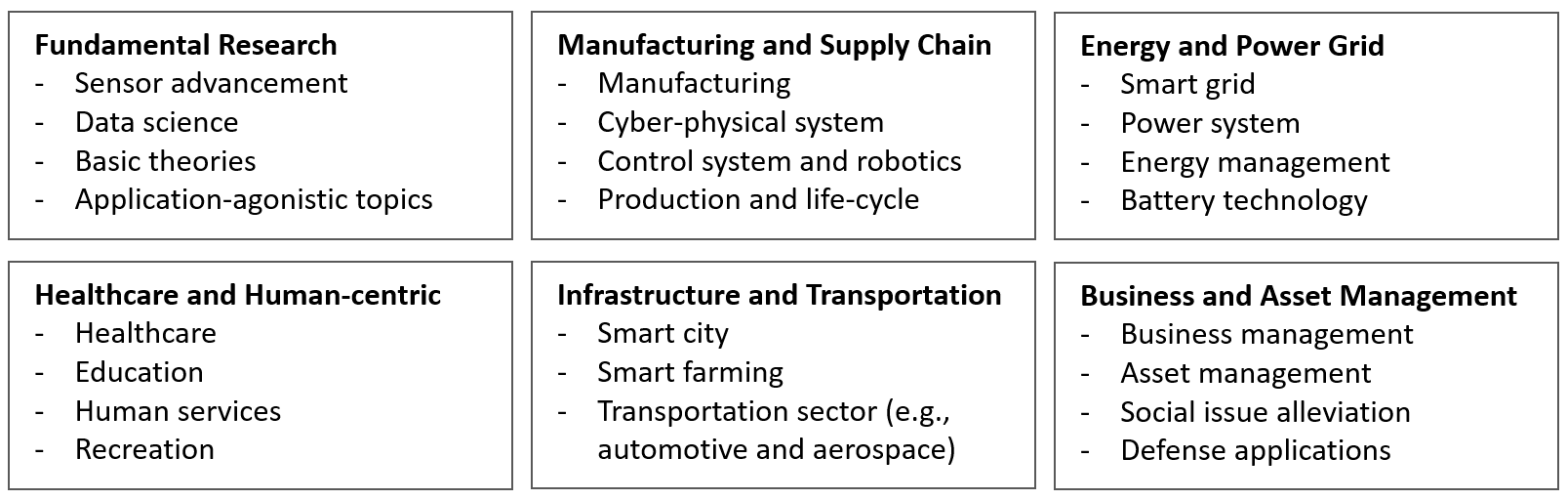
Six primary digital twin application categories.

We classified the papers according to their respective application categories to analyze the research trends of digital twins and their applications in different fields. We identified six primary application categories. Figure 2 displays the subtopics under each primary category. Although a paper may fall under multiple categories, it is assigned to the most relevant category based on its content.

### 2.2. Keyword Co-Occurrence Network Construction

After collecting the metadata from the publications identified in the initial screening, we converted this unstructured data into a structured format suitable for quantitative temporal analysis. To achieve this, we started by extracting keywords and key phrases from the abstract, keywords section, and the title of each paper. We then used a Natural Language Processing (NLP) toolkit to extract essential information while minimizing language biases [26]. The toolkit automatically broke down the title and keyword strings into phrases, eliminated common words such as “a” and “the”, reduced words to their basic form, and reconciled different terminologies referring to the same concept, such as “cyber-physical systems” and “CPS”.

To construct a KCN using the structured data, each keyword is considered as a node, and the co-occurrence of a pair of keywords in the same paper is treated as an edge connecting the co-occurring keyword pair (node pair). The resulting KCN is undirected and weighted, with edge weights indicating co-occurrence frequencies. The KCN, which consists of *n* unique keywords, is stored in an n×n adjacency matrix a. The value of each cell in the matrix, $a_{ij}$, is set to 1 if a connection exists between keyword *i* and *j*, and 0 otherwise.

This study conducts two types of analyses using the KCN. The first type is a temporal research trend analysis, in which we segment publications into distinct time windows: 2000–2020, 2021, 2022, and 2023. We build a separate KCN for each time window to capture the evolving trends over time. The second analysis focuses on capturing research highlights within each application field. Therefore, we divided the publications by their application category and constructed an individual KCN for each category. After constructing the network, we calculated various network metrics for our subsequent analyses.

### 2.3. Network Metrics

This study applies five network metrics to evaluate the KCN. These metrics help identify important keywords, understand their interconnections, and determine the overall structure and trend of the research field. These metrics measure node centrality, connections, and the local topology of node groups.

Node centrality is measured by degree and strength. The **degree** of a node refers to the number of unique nodes it directly connects to. The degree can be calculated using the adjacency matrix a. As shown in Equation (1), the degree of node *i* is the sum of $a_{ij}$. Node *j* belongs to the group of nodes $N_{i}$ that directly connects to node *i*.

(1) $$d_{i}=\sum_{j\in N_{i}}a_{ij}$$

The **strength** of a node counts the number of connections it has, taking into account the frequency of co-occurrences. As shown in Equation (2), the strength of node *i* is a weighted sum of $a_{ij}$, where $w_{ij}$ is the number of connections between node *i* and node *j*.

(2) $$s_{i}=\sum_{j\in N_{i}}a_{ij}w_{ij}$$

**Average weight as a function of endpoint degree** quantifies the relationship between the connectivity of nodes and the strength of their co-occurrences. To calculate this, we define the endpoint degree of an edge connecting node *i* and *j* as $d_{i}d_{j}$. We then examine the relationship between $w_{ij}$ and $d_{i}d_{j}$ for every edge in the network. Due to the multiplicity of edges with identical endpoint degree values in large networks (e.g., 1×50 and 5×10 both equal 50 and hence will have the same endpoint degree), we aggregate edges into set *E* when they share the same endpoint degree. The average weight for edges in set *E* is then calculated by Equation (3), where |E| is the count of edges in set *E*. By plotting ${\bar{w}}_{E}$ against the endpoint degree of edge set *E*, we can visualize the patterns in keyword connectivity. A positive correlation indicates that highly connected keywords (i.e., keywords connected with high strength) tend to co-occur more, while a negative correlation suggests that less connected keywords (i.e., keywords connected with low strength) tend to co-occur more.

(3) $${\bar{w}}_{E}=\frac{\sum_{(i,j)\in E}w_{ij}}{\left|E\right|}$$

The local topology of a network was measured by the average weighted nearest neighbor degree and weighted clustering coefficient. The **average weighted nearest neighbor degree** indicates the strength of a node’s connection with its high- or low-degree neighbors. As shown in Equation (4), the average weighted nearest neighbor degree $d_{i}^{w}$ of a node *i* is obtained by adding up the weighted connections of a node’s direct neighbors and dividing it by the node’s strength. A higher value indicates that a keyword is typically associated with highly connected keywords. By plotting $d_{i}^{w}$ against $d_{i}$, we can visualize how nodes of different degrees behave in terms of connectivity.

(4) $$d_{i}^{w}=\frac{1}{s_{i}}\sum_{j\in N_{i}}a_{ij}w_{ij}d_{j}$$

The **weighted clustering coefficient** measures the level of interconnectedness among a node’s neighboring nodes, taking into account the weight of each connection. As shown in Equation (5), the coefficient $C_{i}^{w}$ is calculated by averaging the weights $w_{ij}$ and $w_{ih}$ of the connections that node *i* shares with its neighbors, node *j* and node *h*. The calculation incorporates a normalization factor $s_{i}\left(d_{i}-1\right)$, which adjusts for the number of potential connections and the strength of each. A high $C_{i}^{w}$ value indicates that a node’s neighbors are not only interconnected but also connected through stronger ties; this suggests a more cohesive and tightly knit structure around the node. In the context of KCN, a high-weighted clustering coefficient for a keyword indicates robust thematic clustering.

(5) $$C_{i}^{w}=\frac{1}{s_{i}\left(d_{i}-1\right)}\sum_{j,h\in N_{i}}\left(\frac{w_{ij}+w_{ih}}{2}\right)a_{ij}a_{ih}a_{jh}$$

## 3. Results

In this section, we first analyze the evolution of digital twins research over time, featuring visualizations of emerging and declining topics. Then, we examine the application of sensor technology across different fields, highlighting principal keywords in each area. Finally, we present specific case studies from each application field, showcasing the practical implementations of digital twin technology.

### 3.1. Research Landscape Evolution over Time

From the KCN analysis results, we see a clear growth and diversification trend in the digital twin field. Table 2 presents the statistics of KCNs from 2000 to 2023. Here, we observe a substantial rise in the number of articles, keywords, and links, particularly after 2020. The increase in articles indicates a surge in research activities, while the growth in links points to an expanding web of interconnected topics.

The development stage of this research field can also be assessed with the *K* value, based on Kuhn’s model of scientific progression [27]. The *K* value is calculated by dividing the number of unique keywords by the frequency of those keywords within a discipline. Derived from Table 2, the *K* values for four different time windows are 0.174, 0.155, 0.119, and 0.105, respectively. The declining trend in the *K* value, inversely proportional to the growing number of publications, suggests that the field of digital twins is in the midst of an evolution, aligning with Kuhn’s pre-revolution or revolution stage.

**Figure 3:**
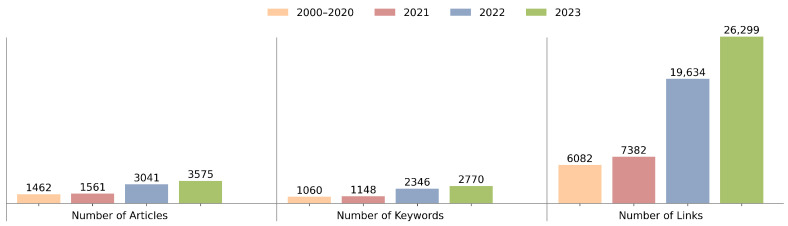
Number of articles, keywords, and links over the four time periods.

**Figure 4:**
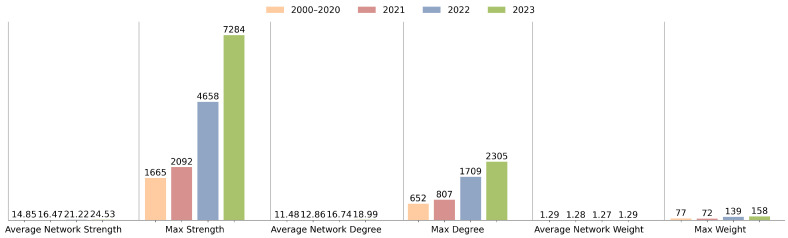
Growth trends in KCN parameters such as average network strength, maximum strength, average network degree, and maximum degree.

Figure 3 reinforces this observation by showing the distribution of articles, keywords, and links across the four time periods, with significant growth in the latter two years. This suggests not only an increase in the research volume but also expansion in the complexity within the field. Figure 4 expands on these data by comparing the average network strength and the maximum weight of the network, both of which indicate an increase in inter-article and inter-topic connections.

**Figure 5:**
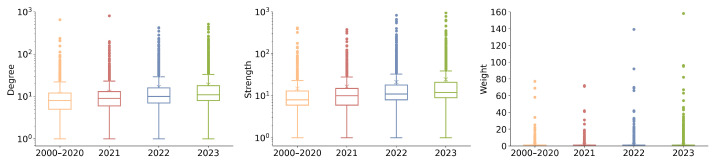
Boxplots of keyword degree, strength, and link weight distribution in the KCN.

Figure 5 offers a distribution of keyword degrees, strengths, and link weights. The upward trends in average and maximum network degrees from Figure 4 and Figure 5 hint at a broadening scope of individual topics and articles, suggesting an increasingly collaborative research environment where topics are more interconnected. Notably, the outliers represent the keywords that are highly connected and centric to this research field. We will present and discuss these topics in the following sections.

**Figure 6:**
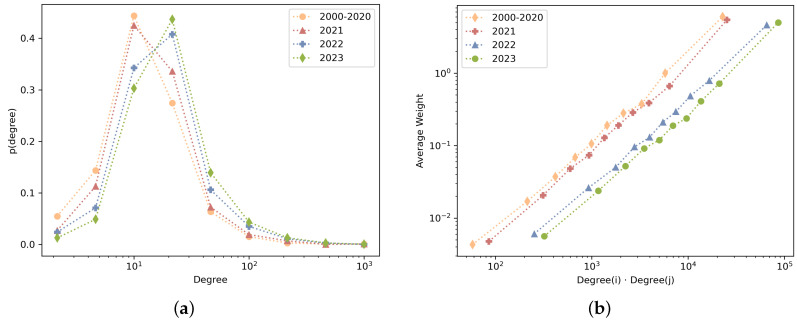

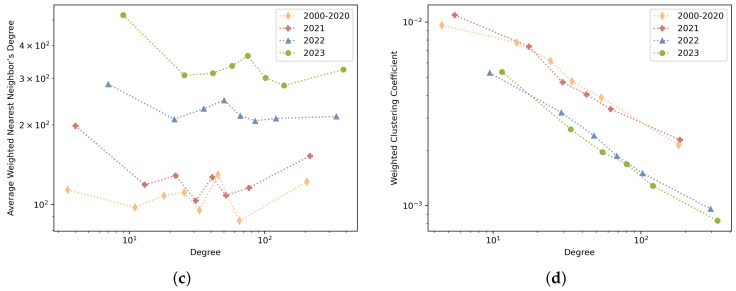
Linecharts of KCN metrics: (**a**) probability density function of keyword degree, (**b**) average weight as a function of endpoint degree, (**c**) average weight as a function of endpoint degree, and (**d**) weighted clustering coefficient as a function of node degree.

Figure 6 provides various insights into the dynamics of the network. Figure 6a shows the probability density function of keyword degree. A shift toward the right over time indicates that certain keywords are becoming increasingly prominent within the network. Figure 6b examines the average weight as a function of endpoint degree. The positive linear trend suggests that keywords with a higher degree tend to form stronger connections with other keywords. However, it is not clear from this graph alone whether these connections tend to be with other highly connected keywords or emerging keywords. In addition, the subtle shift toward the right with time means that the combination of keyword degrees associated with a given average weight has been growing with time, suggesting popular keywords start to be the hubs that connect newer topics into the network, facilitating the network’s growth.

Figure 6c presents the relationship between the average weighted neighbor’s degree and the node degree. In all four time windows, there is no clear correlation between the degree of a node and its neighbor’s degree. This complements the insights from Figure 6b and shows that highly connected keywords connect with a diverse range of nodes rather than only with other highly connected nodes. To accompany this insight, Figure 6d shows the decreasing trend in the weighted clustering coefficient, indicating that highly connected nodes act more as bridges than remaining within isolated clusters, pointing to an expanding and diversifying field.

These visualizations and metrics depict the characteristics of a rapidly growing complex field, with foundational research expanding and certain topics gaining more prominence. However, the consistent average weight across years also suggests that the additional links may not always contribute to the foundational research, raising questions about the depth and influence of recent publications. This nuanced view of the field’s evolution indicates both robust growth and areas requiring further investigation to understand the research impact.

### 3.2. Emerging and Declining Research Topics

**Figure 7:**
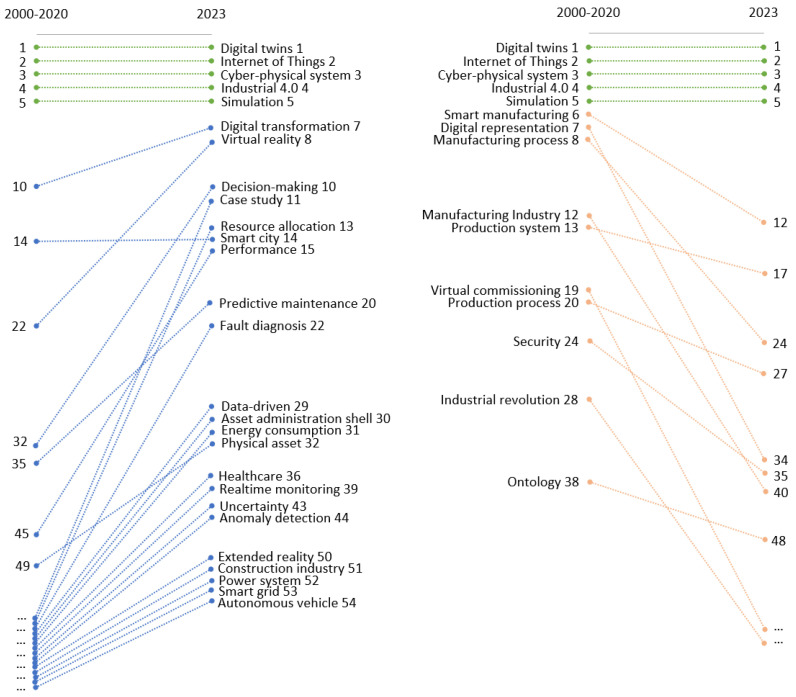
Emerging and declining keywords of digital twin applications from 2000–2020 to 2023.

**Figure 8:**
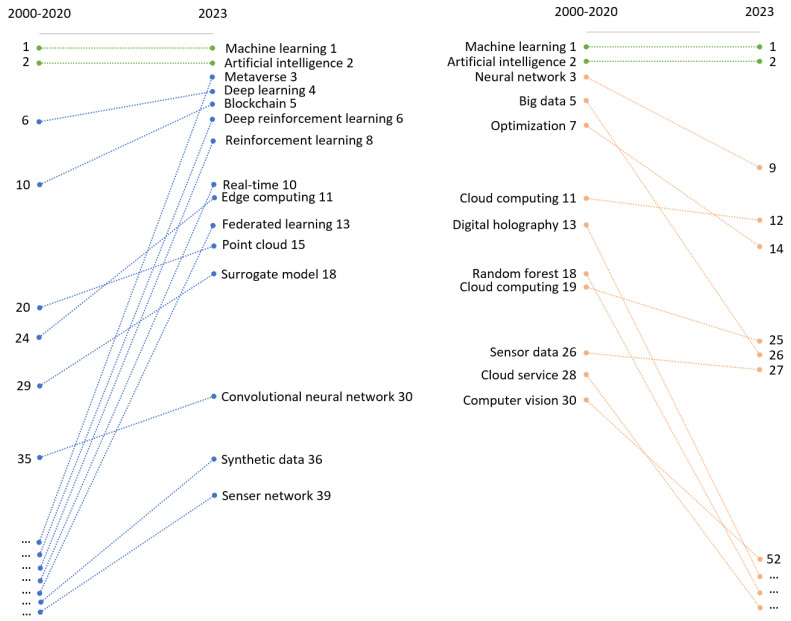
Emerging and declining keywords of digital twins sensing technology from 2000–2020 to 2023.

Figure 7 and Figure 8 trace the changes in keyword relevance over time, from the earliest time window of 2000–2020 to the most recent time window of 2023. In each time window, we ranked keywords based on their strength, which is determined by the number of connections each keyword has. We then compared the ranking of keywords from both time windows to assess emerging or declining trends.

Because there was a substantial increase in overall keyword strength from the earlier to the later time window, we used rank as a proxy for a keyword’s relevance within its specific period. Additionally, we categorized the keywords into two groups: those relating to digital twin applications and those relating to the sensing ecosystem, which includes sensors, machine learning methods, and computational systems.

It is important to note that a slight decline in a keyword’s rank does not necessarily indicate a decrease in its importance or research focus. Instead, it may indicate a natural transition of the keyword from a novel research area to a more established topic that no longer occupies the forefront of emerging research themes. This shift can be seen as a maturation process within the research landscape, where once-novel concepts become foundational elements of the field.

Figure 7 presents keywords related to digital twin applications. Notably, the top five keywords, namely digital twins, Internet of Things, Cyber-Physical Systems, Industry 4.0, and simulation, have maintained the top five positions with no rank change, indicating that they have endured centrality and significance in digital twin research for over two decades. Digital twins, as the literature searching criteria, is naturally included in all research. The Internet of Things is significant for providing the sensor data that feeds digital twins. Cyber-Physical Systems are essential as they constitute the framework in which digital twins operate, integrating computation with physical processes to enable automated decision making. Industry 4.0 represents the current trend of automation and data exchange in manufacturing technologies, including Cyber-Physical Systems, the IoT, and cloud computing, which are inherently linked to the concept of digital twins. In addition, simulation serves as the analytical engine that enables the virtual representation to predict the behavior and performance of its physical counterpart.

There are two types of keywords that indicate emerging trends: application fields (areas where digital twins are being applied) and functions (what digital twins help achieve). The emerging application fields for digital twins include smart cities, energy consumption, healthcare, the construction industry, power systems, smart grids, and autonomous vehicles. The more digitalized and intelligent infrastructure in these areas enables the implementation of digital twins. The increasingly diversified application fields for digital twins also explain the slight decline of smart manufacturing and the manufacturing industry in the right panel.

The emerging functions include digital transformation, decision making, resource allocation, predictive maintenance, fault diagnosis, and real-time monitoring. The trend can be attributed to advancements in machine learning and sensor technologies. As machine learning algorithms have become more sophisticated, digital twins are now able to not only replicate physical systems but also transform and optimize them. Digital twins also have enhanced decision-making capabilities, enabling automated and informed decisions based on predictive analytics and real-time data.

Figure 8 presents keywords related to sensor and machine learning technology. The top two keywords are machine learning and artificial intelligence. The emerging keywords related to sensors are real time, point cloud, and sensor network, highlighting the growing demand for sensors that can deliver immediate, interconnected, and diverse data types. Regarding the computation architecture that supports digital twins and machine learning functions, we notice a rising trend in edge computing and metaverse and a declining trend in cloud computing. This points to a research area pivoting toward distributed computing paradigms, suggesting a move to bring processing closer to the data source for quicker insights. This trend implies that while cloud computing has become a well-established field, the frontier of research is moving toward systems that can handle analytics at the edge of networks.

As for the machine learning-related keywords, emerging models include deep learning, reinforcement learning, federated learning, surrogate models, and convolutional neural networks. This emergence corresponds to the need for sophisticated analytical tools capable of processing complex, multimodal sensor data. These methods are particularly suited to the demands of digital twins, offering enhanced capabilities for privacy preservation and data security.

### 3.3. Mapping Keywords to Application Fields

The previous analysis has focused on the temporal characteristics of the research field and the trends in the relevance of the top keywords. In this section, we will delve deeper and examine how digital twin technology, specifically sensing, machine learning, and computation technologies, are being applied in different fields.

In the methodology section, we mentioned that we classified the literature into six application categories. In this section, we draw insights from each category and visualize the insights using a Sankey diagram. The left column of the Sankey diagram lists the keywords of interest, while the right column represents the application fields. The numbers on the left represent the number of papers that contain the keyword of interest. The number on the right is a summation of all streams of numbers from the left. Since we only selected the top keywords in each category to visualize, the number on the right should not be confused with the total number of papers in each category.

**Figure 9:**
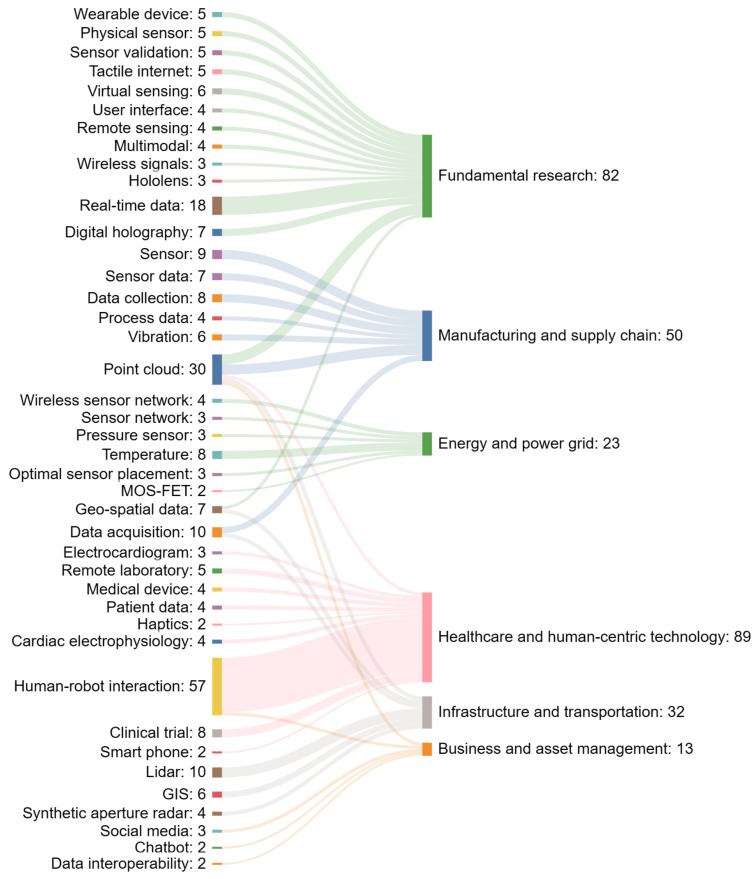
Mapping of sensor technology to digital twin application areas.

Figure 9 displays the mapping from sensor technology to different digital twin application fields. Real-time data and point cloud emerge as the most prevalent keywords, which validates the trend from the slope graph. From this graph, there are two types of sensor keywords: focused sensing technologies and cross-domain technologies. As for focused keywords, process data and vibration have found their place in manufacturing settings as they are common practices for machine and equipment monitoring. Electrocardiograms and cardiac electrophysiology in healthcare may be linked to their potential to create high-fidelity visualizations for cardiac twins. LiDAR shows a strong association with infrastructure applications, likely due to its precision in capturing environmental data for smart city applications.

Cross-domain keywords such as point cloud, data acquisition, and human–robot interaction point toward the versatility of these technologies. Point cloud data, with their high-resolution spatial information, are crucial not only in manufacturing and logistics but also in infrastructure for transportation and urban planning. Data acquisition stands out as a foundational element in the sensing ecosystem to ensure the quality of high-frequency and multimodal sensing data. Human–robot interaction emphasizes the increasing collaboration between humans and automated systems. In healthcare, this could translate to robotic surgery or patient care systems, while, in manufacturing, this can pertain to collaborative robots working alongside human operators.

**Figure 10:**
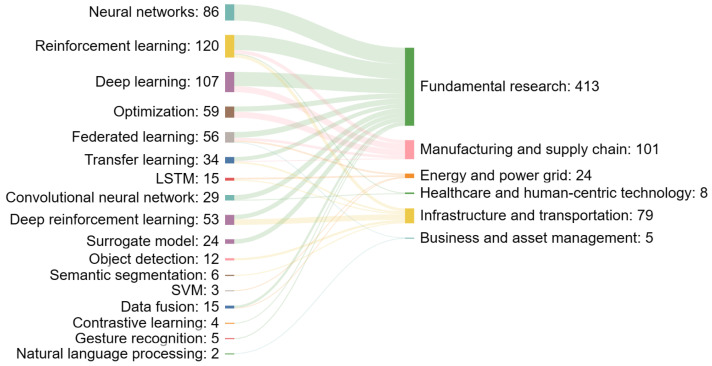
Mapping of machine learning methods to digital twins application fields.

Figure 10 displays the mapping from machine learning methods to different digital twin application fields. Machine learning bestows active digital twins with decision-making capabilities in various applications. Neural networks and deep learning algorithms play a prominent role in pattern recognition and predictive analytics. The strong presence of reinforcement learning, particularly in fundamental research, signals an interest in developing digital twins capable of autonomous decision making and optimization—a critical feature for systems that learn and adapt over time.

The emergence of federated learning points to a growing concern for data privacy and distributed computation, enabling collaborative model training without centralized data storage. This approach aligns well with digital twins, which often require the synthesis of distributed data while sustaining confidentiality, particularly in healthcare and business settings.

The strong connection between optimization techniques and manufacturing and supply chain applications underlines the role of digital twins in process improvement and efficiency gains. Meanwhile, the intersection of convolutional neural networks with infrastructure and transportation highlights their importance in image and video processing tasks relevant to these fields.

Interestingly, the relatively modest numbers attached to healthcare and human-centric technology may reflect the nascent integration of machine learning into these regulated domains, where safety and validation are paramount.

**Figure 11:**
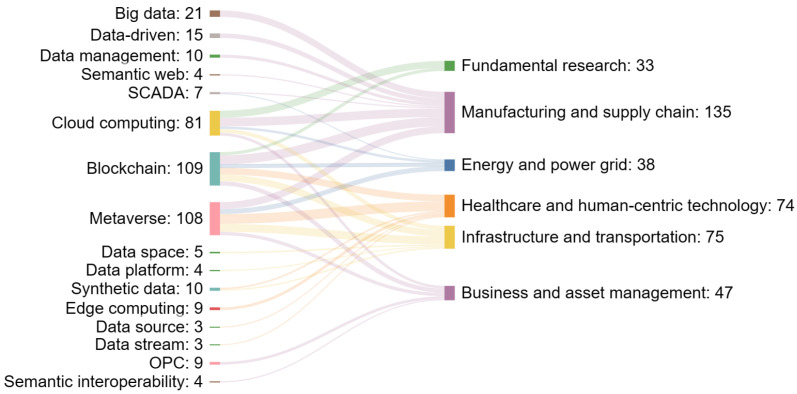
Mapping of computation technology to digital twin application areas.

Overall, Figure 11 displays the mapping from computational technologies to different digital twin application fields. Blockchain’s notable presence across multiple fields, especially in business and asset management, highlights its role in enhancing security, transparency, and traceability. Its application within manufacturing and supply chain domains indicates its potential to revolutionize how data across the digital twin lifecycle are securely managed and shared.

The Metaverse, often associated with immersive virtual environments, shows a substantial intersection with infrastructure and transportation. This could point toward the Metaverse’s capacity for sophisticated simulations and virtual testing environments, which are crucial for planning and managing large-scale infrastructural projects. As suggested by the slope chart above, cloud computing displays a slightly declining influence, indicating a shift toward distributed computing paradigms such as edge computing. Big data and data-driven keywords maintain a steady connection with fundamental research, reflecting the ongoing need to process and analyze large datasets within the digital twin sphere to extract meaningful insights. In addition, semantic interoperability and data fusion, though not as dominant, indicate niche but vital areas in ensuring that digital twins can communicate effectively across systems and synthesize information from disparate sources.

### 3.4. Specific Cases in Each Application Field

Guided by the insights from the previous section, we select and review specific instances of digital twin research in this section. The tables in this section are a curated collection of publications based on the highlighted keywords from our Sankey analysis. This section aims to transition from high-level trends to individual research efforts, providing examples of how sensing, machine learning, and computation technologies are implemented within various application areas.

Table 3 presents a selection of studies in fundamental research of digital twins. The study on sensor calibration within building systems [28] demonstrates the ongoing effort to synchronize physical and virtual sensor data, which is a crucial step for accurate digital twin simulations. Research into sensor reliability [29] tackles the challenge of predictive maintenance by using redundant digital sensors to foresee potential sensor failures. Both studies emphasize the significance of sensor calibration in maintaining the operational integrity of digital twins. Challenges remain in improving the accuracy of a virtual model while maintaining the complex system built upon multiple sensors. Wearable ECG sensors [30] have been studied for low-latency signal analysis, enhancing the responsiveness of digital twins. This research resonates with the need to make digital twins interactive and user-centric. In the future, digital twins will serve not only as tools for simulation and monitoring but also as an end-to-end platform for interaction, providing intuitive feedback to users. The integration of tactile sensors in tactile devices [31] offers insight into the sensory augmentation possibility within digital twins, while the use of LiDAR for user interface design [32] highlights the importance of high-resolution spatial data in creating intuitive teleoperation systems. Both studies suggest that as the digital twin userbase grows, the user experience will become an essential factor, particularly in how objects are identified and interacted with within these virtual environments. Researchers should recognize that the usability of digital twins is as important as their technical accuracy [9].

Table 4 presents a selection of research efforts showcasing the application of digital twin technology in the manufacturing and supply chain areas. In CNC machining, force sensors monitor the cutting torque in end milling processes, supplying data for a comprehensive dashboard that integrates real and simulated torque signals for condition monitoring [35]. The predictive maintenance capacity enables real-time adjustments and machine downtime reduction. Another study develops nonlinear multi-variant dynamic models of multi-axis machine tools with onboard CNC sensing data and visualizes the servo system’s dynamics [36]. The digital twins in the form of real-time visualization can help optimize the machine tool performance and reduce production errors.

Further, in CNC machining, the fusion of tool, workpiece, and process monitoring data, is visualized on a dashboard, providing a complete view of the manufacturing process [37]. This digital process twin supports operators in making informed decisions by simulating part geometry and process analytics. The use of optical sensors in a cyber-physical production cell to create an interactive visual replica [38] signifies the importance of high-fidelity models for understanding and optimizing complex production systems. The above approaches to building digital twin models have made significant progress in unveiling the relations between key indicators and tool performance in the machining process. The sensor-based digital twins allow autonomous monitoring and troubleshooting within smart manufacturing environments. Future work could investigate the scalability of the method to consistently deliver accurate responses and optimize processes as the numbers of machine types and operational parameters scale. Additionally, exploring the integration of machine learning across different manufacturing environments would be valuable.

In additive manufacturing, embedded distributed fiber sensors are used for Finite Element Analysis (FEA) simulations of temperature and strain [39]. The ability to model these parameters with high precision is indicative of the move toward high-fidelity simulations in digital twins, ensuring product quality and process reliability.

Lastly, the production planning process benefits from the fusion of CPS indicators, production data, and LiDAR-generated point clouds to create a 3D model of a production plant [40]. This example demonstrates the potential of digital twins in providing a comprehensive three-dimensional context for production planning, facilitating better spatial understanding and resource allocation.

Table 5 presents the selected applications of digital twin sensor technology in the energy and power grid sector. The energy equipment monitoring example showcases a condition monitoring digital twin of a small hydro turbine, enabled by a wireless sensor network, including accelerometers, temperature, and inductive current sensors. The digital twins operating on sensor readings and environmental data provide a condition indicator visualization [41]. This approach can detect faults early and reduce downtime, which is crucial in the energy sector where continuity is essential.

In electric power conversion, the photovoltaic (PV) dc–dc converter’s efficiency is augmented by thermal cameras and scanning electron microscope imagery. FEM simulations predict temperatures at critical converter components, enabling fast estimations of device conditions under various operational stresses [42]. The two studies highlight the importance of digital twin predictive maintenance in infrastructure reliability.

Wind engineering research utilizes wind pressure sensors to develop an optimal sensor placement algorithm [43]. This algorithm aims to reconstruct wind pressure fields accurately, which is indispensable for assessing the structural integrity of wind turbines and optimizing their design for maximum energy capture. The reconstruction of the wind pressure field can also be utilized for creating digital twin infrastructure.

For hydropower generation, pressure sensors are deployed within the hydraulic network, informing the development of a control system that maximizes hydropower production while adhering to hydraulic constraints [44]. Such digital twin applications ensure the harmonization of power generation with environmental and infrastructural considerations.

Lastly, in the field of smart grids, event loggers are utilized to develop digital twins with autonomous proactive agents [45]. These agents interact within a coordination platform to manage the complex dynamics of energy demand and supply, thus enhancing grid stability and operational resilience. This research points out that integrating an agent-coordination model into digital twins can address complex energy management issues at the microgrid level. The findings provide an example of how to create resilient and user-centric energy networks.

Table 6 presents the applications of digital twin sensor technology in the healthcare and human-centric area, where sensors are broadly referred to as any device or system that detects events or changes in a given environment, transmitting the information to other devices. In the cardiology field, ECG sensors are integral in developing a digital twin of the human heart [46]. This innovative approach merges ECG data with medical records to construct a “Cardio Twin”, a proof of concept that offers heart condition visualizations for both local and remote diagnosis. In another example, clinical 12-lead ECGs and Magnetic Resonance Imaging (MRI) create biophysically detailed digital twins for cardiac electrophysiology [47]. These models simulate intricate heart structures, including Purkinje networks, paving the way for in silico clinical trials and advanced cardiac care.

For rural healthcare, IoT sensors and devices are leveraged to bring medical services to remote areas [48]. Here, sensors encompass a variety of medical devices that collect health-related data, which, when coupled with blockchain technology, ensures secure data management and analysis in resource-limited settings. In space medicine, sensors include mixed reality devices such as HoloLens and haptic systems, which create a digitized interactive training environment [49]. This expands the sensory experience by providing real-time feedback and immersive scenarios for astronaut medical training. The above research highlights the potential and success of digital twin technology in the field of personalized and predictive healthcare.

In the educational sector, sensors refer to the instrumentation of a remote lab, where equipment control and monitoring are critical [50]. These sensor systems enable a hybrid remote laboratory for various learning scenarios, fostering interactive and multimodal educational experiences. This also encourages future researchers to explore digital twin solutions for better learning outcomes and operational safety [52].

Lastly, in the context of human–robot collaboration, force/torque sensors on a battery pack assembly line provide data for a digital twin that visualizes and analyzes the collaborative environment [51]. This digital twin assists in designing, developing, and operating a safe and efficient human–robot interactive system. Future study can revolve around the safety and optimization of these systems with the aid of digital twins [53].

Table 7 presents examples of the use of digital twins in the optimization of infrastructure and transportation systems. For infrastructure modeling, LiDAR sensors are utilized to capture detailed point cloud data of campus buildings [54]. This technology enables the creation of accurate digital replicas of large structures and facilitates the creation of accurate digital twins of extensive structures, enabling efficient maintenance planning and historical preservation.

In transportation infrastructure, the fusion of 2D images from cameras and 3D point clouds from LiDAR leads to a comprehensive digital twin of a magnetic levitation track [55]. This detailed representation bridges the gap between macroscopic project management and microscopic engineering analysis, underscoring the capacity for digital twins to offer multiscale insights into transportation systems. This study points out the importance of having efficient and automated processes for managing large LiDAR datasets to enhance the scalability of digital twins in civil engineering. Future study should include developing advanced algorithms that could automate the conversion of point cloud data into information modeling.

Urban logistics can benefit from the integration of sensors and actuators within the infrastructure. This can be achieved through a platform architecture for digital twins that informs policy making via interactive dashboards [56]. This approach allows for real-time sensor data and logistics system documentation to drive simulation models that can pinpoint gaps and opportunities for transformation within city ecosystems. It is challenging to convert this framework into physical systems for city planners and logistics stakeholders to use and improve urban logistics.

Factory logistics can be revolutionized by incorporating Automated Guided Vehicles (AGVs) that track and monitor the movement of goods on the assembly line [34]. The development and application of a multi-objective AGV scheduling method based on digital twins reflect a shift toward intelligent and efficient logistics systems.

The planning of long-distance freight flows can be analyzed by integrating IoT sensors, GPS, and GIS into a virtual infrastructure and transportation model [57]. This digital twin serves as a powerful tool for analyzing and synchronizing transport, demonstrating the potential of digital twins to streamline logistics operations across vast distances. Future study can focus on achieving interconnection between real-time data and virtual models for different transport modes in an operational context.

The predictive maintenance of agriculture equipment can be improved by digital twin technologies integrated with sensors and data pipeline systems [58]. This study streamlines computational fluid dynamics (CFD) simulation data, sensor readings, and historical information to replicate a virtual cyclone bag filter system in grain milling plants. The digital twins of the system can monitor the filter status and perform precise predictions of a system’s remaining useful life. This research marks the potential of digital twins in improving operational efficiency for smart agriculture through monitoring and predictive analytics.

Table 8 explores the digital twin applications in business and asset management. These sensors are not limited to traditional physical devices but also include digital and social data sources. In production management, the term “sensor” encompasses product documentation throughout production [59]. This documentation acts as a sensor by providing continuous feedback on the product lifecycle, enabling the development of a digital twin for efficient tracking in high-volume production environments. This study set an example of using Asset Administration Shell to standardize and simplify the digital twin representations of manufacturing assets. Another study in this domain proposes a hybrid digital twin approach that integrates traditional onboard sensors with telemetry data sources to create virtual production line properties [60]. Their innovative usage of Apache StreamPipes for handling high-volume data streams features a solution to the data preprocessing for digital twins.

The notion of sensors expands further in environmental monitoring, where social media posts on platforms such as Twitter become inputs. These “digital sensors” capture real-time data on the spread of invasive species [61], offering a novel approach to environmental monitoring by harnessing crowd-sourced information. This study manifests the versatile nature of digital twins by bridging it with ecological management and leveraging Natural Language Processing to model the spread of an invasive species. Additionally, in social issue alleviation and network sentiment analysis, chat rooms and business intelligence data act as sensors by providing communication data and social network dynamics [62,63]. These data allow for real-time sentiment analysis and conversation facilitation via chatbots. The concept of semantic digital twins for simulating human behavior for analytical purposes is an innovative idea. In future studies, it is important to address privacy and data security concerns, such as modeling complex human behavior and ensuring ethical use of personal data.

## 4. Discussion

Our analysis of digital twin research from 2000 to 2023 shows that the field has been growing and diversifying rapidly. This trend, particularly notable post-2020, offers digital twin researchers avenues for new research opportunities and gives digital twin architects insights into the evolving applications in areas such as smart cities, healthcare, and energy. Emerging functions such as decision making and predictive maintenance also demonstrate the field’s advancement.

Sensor-related keywords, such as real time, point cloud, and sensor network, are becoming more important. This emphasizes the demand for real-time, interconnected, and multimodal sensor data. This trend aligns with the computational architecture shift from cloud to edge computing, indicating a move toward distributed computing for faster and more efficient data processing. Moreover, the emergence of advanced machine learning models such as deep learning and federated learning reflects the increasing complexity of sensor data processing. It also highlights the growing importance of privacy and security for digital twins.

According to the keyword trends, digital twins are advancing beyond conventional simulations such as FEA. The research suggests that there is a shift toward developing systems that not only replicate physical entities but also evolve with them. The importance of keywords such as human–robot interaction and predictive maintenance has been growing, which indicates the emergence of interactive and preemptive digital twins.

The Sankey diagrams demonstrate the wide range of sensor technologies used in digital twins. These sensors go beyond traditional physical devices and include medical equipment, clinical tests, and even social media platforms. This approach allows digital twins to provide a comprehensive and accurate representation of real-world scenarios. Moreover, the literature highlights the rise of virtual and soft sensors, which mirror physical sensor functions, offering preemptive insights and facilitating proactive sensing system maintenance.

Our study also revealed a connection between sensor selection and the functionality levels of digital twins, namely representation, replication, reality, and relational. From the review of specific digital twin examples, we observe that the choice of sensors directly influences the level of digital twin functionality, particularly in retrofit designs. As the digital twin capacity increases, we also observe that many applications need sensor data fusion. For example, in the manufacturing setting, a proactive machine tool digital twins will need the input of low-frequency production data and high-frequency sensor data. Incorrect sensor choices can lead to a mismatch between sensor capacity and the expected functionality of digital twins, eventually impacting the performance of the digital twins.

In addition, we believe that relying heavily on data-driven insights without substantial domain expertise can be risky. With limited domain knowledge, there is a possibility of creating brittle and underwhelming digital twins that may not respond inadequately to real-world variables. Therefore, future research should aim at integrating domain knowledge and robust empirical knowledge into digital twins to enhance their reliability and accuracy.

## 5. Conclusions

This study analyzed the research trends in the field of digital twins by examining metadata from 9639 peer-reviewed articles published between 2000 and 2023. We processed the metadata using an NLP-based toolkit and manually labeled each article with its most relevant application field. Using the KCN methodology, we performed temporal research trend analysis, mapping popular sensing technologies to six application fields and identifying representative examples of digital twins in each field. For researchers, this analysis provides a comprehensive view of the field’s development, identifying key areas for future exploration. For architects, the findings highlight technological applications and examples essential for informed decision making in digital twin system design.

This study found that the field of digital twins is rapidly growing and diversifying. We used network metrics to analyze the temporal changes in the field and identified emerging and declining keywords over time. We also identified emerging application fields, functions, and enabling sensing technologies. The findings suggest that digital twins are moving toward predictive tasks while ensuring system integrity and security across many sectors beyond manufacturing.

We used a Sankey chart to visualize the mapping from popular sensing technologies to six application fields. We found that real-time data, point cloud data, and human–robot interaction are increasing trends. Additionally, we noticed an extension of the traditional sensor definition to include novel sensors such as medical tests and social media posts. We identified neural networks and reinforcement learning as crucial for autonomous decision making. The emergence of federated learning marks a shift toward distributed computation, emphasizing data privacy.

Following the mapping, we reviewed specific examples of digital twins in each field. For each application, we analyzed its physical assets, sensors, physical–digital data flow, the form of digital assets, and research objectives. From these examples, we observed a connection between sensor selection and the functionality level of digital twins. We raised concerns over the mismatch between sensor capacity and digital twins’ functionality and possible brittle digital twins if they are too dependent on empirical prior knowledge.

## Figures and Tables

**Table 1:** A selection of digital twins review papers.

Application Area	Key Contributes	Gaps/Opportunities Identified	Ref.
General Applications	Reviews covering concepts, key enabling technologies, and implementation of digital twins, including challenges and prospects across multiple domains	The need for standardization, data availability, processing power, interdisciplinary collaboration, and development of reference frameworks and performance metrics	[12,13,14,15]
Smart Manufacturing	Reviews digital twins integration in Industry 4.0 and digital supply chains and their optimization potential	The need for design frameworks, early detection of design flaws, clarity in research focus, and organized research environment	[16,17]
Smart Grid and Smart City	Review of digital twins in energy management and infrastructure durability	Challenges in data management, analysis, real-time interaction, and effective distributed sensing updating	[18,19]
Agriculture	Current trends, roadmap, and open questions in digital twins for agriculture	The need for automated decision-making support and complex examples	[20,21,22]
Smart Healthcare	Review of digital twin applications in precision medicine, clinical trial design, hospital operations, and platforms supporting mobile health applications	Technical, regulatory, and ethical challenges in healthcare digital twins	[23,24]
Education	Review of digital twins in remote and virtual laboratories	Integration of digital twin concepts into educational systems	[25]

**Table 2:** Temporal analysis of four KCNs from four time periods.

Metric	2000–2020	2021	2022	2023
Number of Articles	1462	1561	3041	3575
Number of Keywords	1060	1148	2346	2770
Number of Links	6082	7382	19,634	26,299
Average Network Strength	14.85	16.47	21.22	24.53
Max Strength	1665	2092	4658	7284
Average Network Degree	11.48	12.86	16.74	18.99
Max Degree	652	807	1709	2305
Average Network Weight	1.29	1.28	1.27	1.29
Max Weight	77	72	139	158

**Table 3:** Examples of articles covering fundamental research in digital twins.

Domain	Physical Asset	Sensors	Physical–Digital Data Flow	Form of Digital Asset	Research Objective	Ref.
Sensor calibration	Building system and sensors	Temperature and pressure sensors	Sensor readings	Building automation system (BAS) interface	Simultaneous in situ calibration of physical and virtual sensors	[28]
Sensor reliability	Sensors	Vibration, temperature, and force sensors	Sensor readings	Simulated readings of the redundant digital sensors	Predict sensor failure using redundant digital sensors	[29]
Sensor connectivity	Human	Wearable ECG sensor	Sensor readings	Low-latency signal visualization and analysis	Enhanced biosensor data flow across a Bluetooth to Ethernet gateway	[30]
Tactile sensing	Tactile device	Tactile sensors	Touch events	Across-platform semantic abstraction and visualization	Ontology model construction for tactile sensing devices	[31]
Data processing	Truss	Force sensor and strain gauge	Sensor readings	Rapid visualization of the truss stress field	Multi-fidelity surrogate model development	[33]
Scene construction	Bridge construction site	Cameras	Multi-resolution images	Holographic scene in a prototype interface	Dynamic holographic modeling approach for augmented visualization of digital twin scenes	[34]
User interface design	Geometrical objects	Onboard robot sensing (e.g., LiDAR)	Point cloud	Visualization of the physical asset	Evaluate the recognition of physical objects and its implications on UI design for teleoperation systems	[32]

**Table 4:** A sample of articles covering manufacturing and supply chain digital twin research.

Domain	Physical Asset	Sensors	Physical–Digital Data Flow	Form of Digital Asset	Research Objective	Ref.
CNC machining	Cutting torque in end milling	Force sensors	Process parameters and database of historical torque signals and analysis	Dashboard of simulated and real cutting torque and analysis	Machine tool condition monitoring	[35]
CNC machining	Servo system of a 5-axis laser drill	Onboard CNC sensing	In-process CNC data (e.g., body motion, actuator ripples, and vibration modes)	Real-time visualization of servo dynamic models	Develop nonlinear multi-variant dynamic models of multi-axis machine tools	[36]
CNC machining	Milling machine	Onboard CNC sensing (e.g., torque current and tachometer)	Fusion of tool, workpiece, and process monitoring data	Visual dashboard of part geometry, process data, and analysis	Development of digital process twin	[37]
Cyber-Physical System	CTTP 4.0 production cell	Optical sensors	Fusion of real-time operational parameters and sensor readings	Interactive visual replication of production cell	Practical implementation of digital twin complied to industry standards	[38]
Additive manufacturing	Temperature and strain profiles	Embedded distributed fiber sensors	Fusion of process parameters and sensor readings	FEA simulation of temperature and strain	Model temperature and strain with embedded distributed fiber sensors	[39]
Production planning	Body-in-white (BIW) production system	Onboard sensors and LiDAR	Fusion of CPS indicators, production data, and point cloud of the plant	3D production plant model with optimized production planning	Demonstrate the automated creation and updating of a BIW production digital twin	[40]

**Table 5:** A selection of research articles covering energy and power grid digital twins research.

Domain	Physical Asset	Sensors	Physical–Digital Data Flow	Form of Digital Asset	Research Objective	Ref.
Equipment monitoring	Small hydro turbine	Wireless sensor network (e.g., accelerometers, temperature, and inductive current sensors)	Sensor readings, production, and environmental data	Condition indicator visualization and predictive analysis	Build digital twin from various data source for turbine condition monitoring	[41]
Electric power converter	Photovoltaic (PV) dc–dc converter	Thermal camera	Scanning electron microscope (SEM) images, converter tech specs, and temperature data	FEM simulation and prediction of junction, case, and heat sink temperatures	Fast estimation of switching device temperature in PV converters	[42]
Wind engineering	Wind pressure field	Wind pressure sensors	Sensor readings	Wind pressure field visualization and measurements	Develop an optimal sensor placement algorithm to reconstruct wind pressure fields	[43]
Hydropower generation	Hydraulic network (e.g., valve, tank, and pipe)	Pressure sensors	Hydraulic model of the plant and the valve and flap configuration	Hydraulic variables and hydropower generation visualization and analysis	Develop a control system to maximize hydropower production while meeting hydraulic constraints	[44]
Smart grid	Smart grid (e.g., consumer, producer, and regulator)	Event logger	Live semantic annotation (LSA) of events and coordination laws that cause the events to evolve	Autonomous proactive agents on a coordination platform CLEMAP	Develop a series of digital twins that interact and coordinate activities to exchange energy and enhance grid stability	[45]

**Table 6:** A selection of research articles covering healthcare and human-centric digital twin research.

Domain	Physical Asset	Sensors	Physical–Digital Data Flow	Form of Digital Asset	Research Objective	Ref.
Cardiology	Human Heart	ECG sensors	ECG data and medical records	Heart condition visualization and analysis available for local and remote diagnosis	Proof of concept version of the Cardio Twin	[46]
Cardiology	Human heart and cardiac electrophysiology	Clinical 12-lead ECG and magnetic resonance (CMR) imaging	ECG data and CMR imaging	Biophysically detailed cardiac twin including Purkinje networks and cardiac electrophysiology	Create personalized, multiscale biventricular heart models for in silico clinical trial	[47]
Rural health	Patients in rural areas	Healthcare IoT sensors and medical devices	Sensor readings	Blockchain-encrypted medical data and analysis	Integrate healthcare IoT data with blockchain for secure and efficient data management in rural healthcare	[48]
Space medicine	Medical training environment	Mixed reality (e.g., HoloLens) and haptic devices	Digitized real-world training scenarios and learner’s real-time input	Interactive training with virtual feedback integrated into real-world scenarios	Develop a mixed-reality-based medical training platform for astronauts	[49]
Education	Remote lab of a production cell	Onboard sensors for equipment control and monitoring	Sensor readings	Interactive interface with multimodal visualizations	Apply the digital twin concept in a hybrid remote laboratory for various learning scenarios	[50]
Human–robot collaboration	Battery pack assembly line	Force/torque sensor	Fusion of sensor readings and production data (e.g., idle times)	Visualization and analysis of the human–robot collaboration assembly line	Design, develop, and operate an agile, adaptable, and safe human–robot collaborative system	[51]

**Table 7:** A selection of infrastructure and transportation digital twins research.

Domain	Physical Asset	Sensors	Physical–Digital Data Flow	Form of Digital Asset	Research Objective	Ref.
Infrastructure modeling	Campus buildings	LiDAR	Point cloud data	Virtual replicas of large campus infrastructure	Assess and implement reconstruction methods to create digital twins of large infrastructure using point cloud data	[54]
Transportation infrastructure	Magnetic levitation track	Cameras and LiDAR	Fusion of 2D images 3D point clouds	Detailed digital representation with macroscopic to microscopic perspective analysis	Fuse 2D image and 3D point cloud to create a digital twin model of magnetic levitation track	[55]
Urban logistics	Urban infrastructure and logistics systems	Sensors, actuators, and logistic system documentation	Sensor readings reflecting the state of the logistic system	Interactive dashboards with metrics and logs to inform policy making	Propose a platform architecture for digital twins in urban logistics, addressing gaps in simulation model orchestrations and data transformation	[56]
Factory logistics	Assembly line with Automated Guided Vehicles (AGVs)	Sensors for AGV tracking and monitoring	Sensor readings and documentation of assembly logistics	Data model, simulation analysis, and virtual action model of the assembly line	Develop and apply an AGV multi-objective dynamic scheduling method based on digital twins to improve logistics efficiency	[34]
Transportation planning	Long-distance freight flows with various transport modes	IoT sensors, GPS, and GIS	Real-time data inputs from transport modes	Virtual infrastructure visualization and transportation mode analysis	Explore the potential of digital twins in synchromodal transport for long-distance freight flows	[57]
Smart agriculture	Cyclone bag filter in grain milling plants	Pressure sensors and anemometer	Real-time sensor readings	Computational fluid dynamics simulation of cyclone bag filter for RUL prediction	Apply digital twins for predictive maintenance of the cyclone bag filter system	[58]

**Table 8:** A selection of business and asset management digital twins research.

Domain	Physical Asset	Sensors	Physical–Digital Data Flow	Form of Digital Asset	Research Objective	Ref.
Production management	Products and assets	Product documentation throughout production	Data from production assets, plants, and operators	Asset Administration Shell (AAS) for lifecycle tracking	Develop a digital twin for efficient lifecycle tracking in high-volume production	[59]
Production management	Steel production line	Onboard sensors and telemetry data sources in production line	Sensor readings, batch data, and manually-input data	Virtual production line properties via the digital twin API	Propose a hybrid digital twin approach integrating Industry 4.0-compliant digital twins with AAS and Apache StreamPipes	[60]
Environment monitoring	Spread of invasive species	Social Media (Twitter)	Posts on spotted lanternfly sightings and behaviors	Visualization of invasive species spread	Create a digital twin using social media content for environmental monitoring	[61]
Social issue alleviation	Social subjects (e.g., personal life, organizations)	Chat rooms	Communication data from chat rooms	Real-time sentiment analysis and Chatbot-driven conversation facilitation	Use chatbots to stimulate and sustain communication in social environments	[62]
Social network	Social sentiments	Business intelligence data infrastructure	Data from social networks	Knowledge graphs for social data mapping	Create semantic-driven digital twins for analyzing social network dynamics	[63]
